# Civilization and Disease

**DOI:** 10.5195/jmla.2019.599

**Published:** 2019-01-01

**Authors:** Melanie J. Norton

**Affiliations:** Head of Access and Delivery Services, Cushing/Whitney Medical Library, Yale University School of Medicine, New Haven CT, melanie.norton@yale.edu

First published in 1943, the classic and oft-cited historical work, *Civilization and Disease*, by Henry E. Sigerist, has been reprinted this year as a paperback by its original publisher, Cornell University Press. The contents remain unchanged from the original with the exception of a foreword added by Elizabeth Fee, “The Life and Work of Henry E. Sigerist.” Fee is the former chief of the History of Medicine Division of the National Library of Medicine at the National Institutes of Health in Washington, DC. It is fitting that she has written the foreword as she is a devoted supporter of Sigerist’s beliefs in universal health care and an active member in the Sigerist Circle.

To better understand and appreciate the contents of the book, it is important to understand Sigerist’s background, and Fee provides a succinct biographical sketch of his experiences, beliefs, and accomplishments. For those readers who are unfamiliar with the man, Fee’s foreword paints a portrait of a man with a brilliant, inquisitive mind and a prolific output including 27 published books and 455 research papers. Not merely a medical scholar, he was a generalist—well-read in many fields—and was a popular and inspiring speaker. Fee writes: “On January 30, 1939, *Time* magazine published his portrait on the cover and included an enthusiastic article describing him as the world’s greatest medical historian and the nation’s most widely respected authority on health insurance and health policy” (p. ix).

Sigerist was born in Paris, France, in 1891 and received his medical degree in Zurich, Switzerland, in 1917. His passion, however, became the history of medicine, which he taught at the University of Zurich until 1925. In 1932, on an invitation from Johns Hopkins University’s founding dean, he moved to the United States and became director of their Institute of the History of Medicine.

Although initially impressed with the rapid advance of American medicine, during this time, he became enamored with the Soviet Union’s model of socialized medicine. After a visit to the Soviet Union, he grew more convinced about the superiority of their system, and in 1937 he wrote, *Social Medicine in the Soviet Union*, a book that supported the idea of compulsory health care coverage administered by the government. Although favorably received by some left-leaning Americans, his views naturally drew the ire of many others who cast him as too pro-communism and even subversive. Facing increasing resentment in American society, he moved back to Switzerland in 1947 and died ten years later (p. xii).

*Civilization and Disease* is based on a series of six lectures Sigerist gave at Cornell University in 1940. Sigerist organized them into twelve chapters that cover disease in such wide-ranging topics as diet, famines, housing, water supply and sewage, work, social life, the law, and religion—all written in a lucid, easy-to-read manner that would be engaging and fascinating to the expert and layperson alike. For example, after discussing societal taboos, public health laws, worker’s comp, forced sterilization, and abortion in his chapter on “Disease and the Law,” he writes an enlightened essay on the quandary of mental illness and crime that could have been written yesterday.

While Sigerist’s views regarding universal health care were rejected by many Americans as too leftist, his ideas were influential in helping to create Canada’s national health care system [1]. His supporters also created the Sigerist Society, which was established by a group of Marxist doctors and was active from 1947–1955. In 1990, after a meeting of the American Association for the History of Medicine in Baltimore, the Sigerist Circle was established. Fee is a member and was once the president of this organization. At a workshop held in April 2018, in Columbus, Ohio, the Sigerist Circle focused on the topic, “Medical Historians Respond to the Age of Trump.” Perhaps it is because of the current political climate that this book was reprinted with a new and revealing foreword.
